# Stress as a Risk Factor for Informal Caregiver Burden

**DOI:** 10.3390/healthcare13070731

**Published:** 2025-03-26

**Authors:** Elena Cejalvo, Manuel Martí-Vilar, Júlia Gisbert-Pérez, Laura Badenes-Ribera

**Affiliations:** 1 Department of Basic Psychology, Faculty of Psychology and Speech Therapy, Universitat de València, 46010 Valencia, Spain; ecehe@alumni.uv.es; 2 Department of Behavioural Sciences Methodology, Faculty of Psychology and Speech Therapy, Universitat de València, 46010 Valencia, Spain; julia.gisbert@uv.es (J.G.-P.); laura.badenes@uv.es (L.B.-R.)

**Keywords:** structural equations, informal caregiver, caregiver burden, family, functional diversity

## Abstract

**Background/Objectives:** Informal caregivers, who provide essential support to dependent family members, often face high levels of stress and mental health problems due to the physical and emotional demands of the role. This study examined the connections between anxiety, depression, stress, and caregiver burden in informal caregivers. **Methods**: A total of 344 informal caregivers from the Valencian Community, selected by incidental sampling, took part. A total of 58.7% were female (*n* = 202) with a mean age of 46.37 years (*SD* = 14.79), while 41.3% were male (*n* = 142) with a mean age of 46.37 years (*SD* = 14.78). This study used the DASS-21 and the Zarit Burden Questionnaire. Data analysis was by structural equation modeling with latent factors. **Results**: The principal findings indicated that anxiety and depression were predictive factors for stress (*p* = 0.006, *p* = 0.002 respectively), while stress, in turn, was associated with a higher caregiver burden (*p* < 0.001). **Conclusions**: Anxiety and depression indirectly heighten caregiver burden through increased stress.

## 1. Introduction

The role of informal carers of people with functional diversity has now gained significant relevance in the health and well-being field. Informal caregivers, also known as family cares or non-professional carers, play a crucial role in providing support and daily assistance to family members with diverse care needs due to conditions of functional diversity [1].

The World Health Organization defines informal caregivers as “people who care for family members, friends or neighbors who need help because of health status, advanced age, disability or frailty” [2]. This definition highlights the broad spectrum of responsibilities and challenges faced by informal caregivers in their daily activities.

The importance of informal carers lies in their ability to provide continuous and personalized care in the home, significantly contributing to the quality of life and well-being of people with functional diversity. They are also recognized as key players in the care system who play an indispensable role in optimizing the access of the disadvantaged to community and health care services, promoting greater efficiency and timeliness in the provision of these services [3].

However, this role brings with it a number of physical, emotional, financial, and social challenges that can impact the health and well-being of both the caregiver and the care recipient [4]. Not the least of these challenges is that caregiving inevitably involves observing the deterioration and anticipatory bereavement of a loved one, which in turn can affect the caregiver’s health [5].

### 1.1. Risk Factors of the Caregiver Burden

Considered to be a distressing condition, anxiety can manifest symptoms that affect daily life, interfering with caregiving tasks and generating a negative impact on both the caregiver and the recipient [4]. Depression, characterized by sadness, loss of interest, and other symptoms, is closely linked to caregiving, especially when the severity of the patient’s disability correlates with an increased risk of depression in the caregiver [6]. The relationship between caregiving, anxiety, and depression is a consistent theme in research, with evidence of carers suffering higher levels of anxiety than the general population [7,8,9]. Stress, conceptualized as an organism’s response to challenging environmental demands, is intrinsically linked to the caregiving experience. Influenced by primary stressors and the caregiver’s subjective appraisal, stress manifests itself as a central component of caregiving [10,11]. Factors such as the intensity and duration of caregiving, lack of social support, financial difficulties, and lack of adequate resources can exacerbate stress, leading the caregiver to experience overload. In addition, the caregiver’s perception of their ability to manage the demands of caregiving, as well as the presence or absence of effective coping strategies, play a crucial role in how stress manifests and is managed [12,13]

The caregivers’ burden, defined as the subjective experience of caregiving demands, is one of its main symptoms. This concept, which has been developed since the 1960s, includes both objective and subjective dimensions and reflects changes and emotional reactions to caregiving demands [14,15]. Research has shown that as the caregiver burden increases, the carers’ physiological and psychological health is negatively affected [16].

Caregivers are thus often referred to as ‘invisible patients’ due to the high levels of the burden, social isolation, and financial problems they face, which can have a negative effect on both their mental and physical well-being [17].

### 1.2. Other Socio-Demographic Variables Influencing Caregiving Status

Research has shown that long hours of caregiving and the severity of the recipient’s functional diversity can significantly increase the carer’s stress and emotional burden. This can lead to mental health problems such as anxiety and depression, as well as physical problems including chronic fatigue and lowered immune systems [18]. As other studies have shown that the negative reactions associated with the caregiving experience may be linked to factors such as the time spent caring, patient deterioration, dependency in daily activities, and disease recurrence [14], these variables were considered for analysis in the present study.

### 1.3. The Present Study

**Figure A1:**
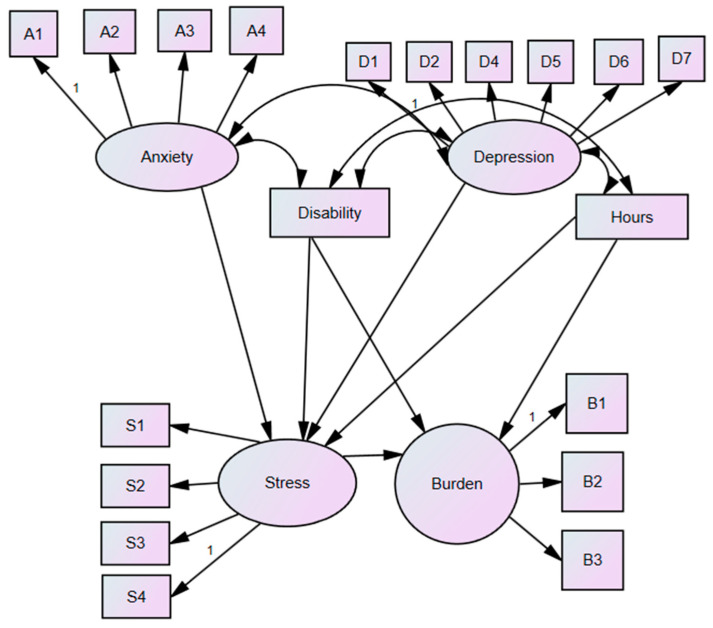
Theoretical proposed model. A: anxiety indicators; D: depression indicators; S: stress indicators; B: burden indicators.

Our aim was to use structural equation modeling to study how anxiety and depression can predict stress (see Figure A1) in addition to exploring how stress acts as a risk factor for caregiver burden. This study seeks to delve deeper into these interactions through the use of structural equation modelling (SEM), which allows for a more comprehensive and multidimensional analysis of these constructs. Unlike previous research that has addressed these variables in isolation or in part, our approach integrates multiple pathways of influence, facilitating a deeper understanding of how these factors interrelate and jointly contribute to caregiver burden.

In addition, this study incorporates key contextual variables, such as caregiving hours and the degree of disability of the care recipient, which have been shown to be critical determinants of the caregiver experience. This study is grounded in the well-established premise that anxiety and depression are significant predictors of stress in caregivers. Extensive prior research has consistently demonstrated that caregivers experiencing higher levels of anxiety and depression are more likely to report elevated stress levels. Escobedo-Aedo et al. [19] found that anxiety and depression not only coexist but also act as key drivers of stress in informal caregivers, particularly in those providing long-term care to individuals with functional diversity. Similarly, Pinquart and Sörensen [20] highlighted in their meta-analysis that emotional distress, including symptoms of anxiety and depression, significantly contributes to the perception of stress in caregiving contexts.

While this relationship is well documented, our study builds on this foundation by integrating these predictors into a more comprehensive model that includes additional contextual variables, such as the hours of caregiving and degree of disability. This allows us to explore how anxiety and depression interact with other factors to influence stress levels, providing a more nuanced understanding of the caregiving experience.

For this, we formulated the following working hypotheses:

**Hypothesis** **1:** *Stress can be expected to act as a risk factor for caregiver burden, so that the greater the stress, the greater is the caregiver burden*.

**Hypothesis** **2:** *There is a positive relationship between anxiety and depression*.

**Hypothesis** **3:** *The level of disability and hours of caregiving can be expected to influence the model’s endogenous endpoints*.

## 2. Materials and Methods

### 2.1. Participants

A convenience sample of 344 Spanish informal caregivers with an average age of 46.35 years (median = 52, *SD* = 14.53, ranging from 19 to 90 years) was recruited between 15 and 30 December 2024. The participants were intentionally chosen through the research team’s contacts and various care centers for dependent individuals in the Valencian Community. The inclusion criteria were as follows: (1) being an informal caregiver of a dependent person, (2) being at least 18 years old, (3) providing informed consent, and (4) residing in the Valencian Community.

Of the participants, 58.7% were female (*n* = 202) with an average age of 46.37 years (*SD* = 14.79), and 41.3% were male (*n* = 142) with an average age of 46.37 years (*SD* = 14.78). In ethnic terms, 90.7% identified as Caucasian, with only 4.4% from other ethnic backgrounds. The educational qualifications varied, with 2.9% having no formal education, and 31.7% possessing a university degree. Regarding marital status, most of the participants were married (54.1%), while 18.6% stated that they were in a relationship. The majority of caregivers (86.6%) provided between 1 and 56 h of care, while 4.4% spent between 57 and 112 h, and 9% between 113 and 168 h. This indicates that the care burden varies, but fewer hours predominate. This study also shows the distribution of disability levels among people cared for by informal carers. Some 17.4% of these people have a mild disability, while 50.9% have a moderate disability. Finally, 31.7% face a severe disability, reflecting the diversity of care needs within this group.

### 2.2. Instrument

A two-section online survey was conducted through the LimeSurvey platform: the first section gathered socio-demographic information (refer to Table 1), and the second comprised various questionnaires designed to evaluate the key variables under study, including anxiety, depression, stress, and the subjective burden.

To measure anxiety, depression, and stress, we used the Depression Anxiety Stress Scales (DASS-21 [21], Spanish version [22], consisting of 21 items with 7 items per subscale: DASS-D (depression) (e.g., I could not feel any positive feelings), DASS-A (anxiety) (e.g., I realised that my mouth was dry), and DASS-S (stress) (e.g., I had a hard time releasing the tension). The respondents were asked to rate the extent to which each statement could be applied to them during the previous week on a Likert-type response scale ranging from 0 (did not apply to me at all) to 3 (applied to me a lot): “Please read the following statements and circle a number (0, 1, 2, 3) that indicates to what degree this statement has happened to you in the past week”.

Since the DASS-21 is a shortened version of the DASS (42 items), the final score for each subscale is the sum of the scores of the items in the scale. A higher score on the scale indicates a higher level of anxiety, depression, and stress. The instrument in its original version showed an internal consistency as follows: depression (α = 0.91), anxiety (α = 0.84), and stress (α = 0.90). In the present study, the internal consistency was adequate: depression (α = 0.860), anxiety (α = 0.833), and stress (α = 0.836).

The Zarit Burden Interview Short Form was used to measure the subjective burden. (ZBI-7 [23], consisting of 7 items (e.g., Do you feel that, because of the time you spend with your relative/patient, you no longer have enough time for yourself?)). The response format of the 7 items was as follows: (1: Never; 2: Almost never; 3: Sometimes; 4: Frequently; 5: Almost always). The total scores were the sum of the items that make up the scale, with higher scores indicating a higher burden. The internal consistency was found to be adequate (α = 0.929).

### 2.3. Procedure

This study adhered to the principles outlined in the 1964 Declaration of Helsinki, including subsequent amendments and comparable ethical guidelines, and received the approval of the Ethics Committee. Prior to the main data collection, a pilot test was carried out to ensure that all items in the questionnaire were clearly understood by the researchers conducting this study and by a small group of participants. This step helped to refine the questionnaire and confirm its clarity and appropriateness for the target population. The questionnaires were distributed online in December 2024, with each session taking approximately 35 min. A link was generated to share the questionnaire with caregivers across Spain. All the participants, who were caregivers of dependent individuals at various centers in the Community of Valencia, provided their informed consent before taking part in this study. To minimize selection bias, it was ensured that the sample included caregivers from different centers and levels of dependency in the Valencian Community. In addition, a randomized control of the sequence of the scales in the questionnaire was implemented to reduce the effect of fatigue or response bias. Participation was entirely voluntary, and the consent form specified the study’s conditions and guaranteed that the participants’ data would be completely anonymous. No incentives were offered to the participants.

### 2.4. Data Analysis

The means and standard deviations were used to describe continuous variables, while frequencies and percentages were used for the categorical variables. Univariate skewness and kurtosis were examined to assess the normality of the data. The Cronbach’s alpha coefficient was calculated to determine the internal consistency of the test scores, with a value above 0.60 being considered satisfactory [24]. A bivariate correlation analysis was performed to assess the strength and direction of the relationships between the variables under study. The statistical analyses were carried out on IBM SPSS software, Version 26.0 for Windows.

Structural equation modelling (SEM) was used to test the study hypotheses, an approach that allowed us to simultaneously examine all the proposed hypotheses, the inclusion of multiple dependent variables along with their measurement errors, and the correlation between variables or their errors [25], following the guidelines proposed by Medrano and Muñoz-Navarro [26]. The maximum likelihood method was used after addressing outliers and missing data, with a previous assessment of normality. Item parceling was used to select indicators of anxiety, depression, stress, and caregiver burden, as follows:

The Zarit Burden Interview measures the burden in three domains: “B1” overload (items 2, 5, and 7), “B2” loss of self-care (items 1 and 4), and “B3” loss of role (items 3 and 6) [23]. Anxiety was measured with the following: A1 (autonomic arousal), A2 (skeletal musculature effects), A3 (situational anxiety), and A4 (subjective experience of anxious reason) [18]. Stress was assessed as follows: S1 (difficulty relaxing), S2 (nervous arousal), S3 (irritable/over-reactive), and S4 (impatient) [21]. Depression was investigated with the following items: D1 (dysphoria), D2 (hopelessness), D3 (devaluation of life), D4 (self-deprecation), D5 (lack of interest), D6 (anhedonia), and D7 (inertia) [21].

The level of disability and hours of care were included as observed variables, while the remainder were treated as latent variables. The level of disability and hours of care were considered exogenous variables, stress and caregiver burden as endogenous variables, and caregiver burden was the final endogenous variable.

Model fit was assessed using the fit indices recommended in the literature: X^2^/df, the comparative fit index (CFI), the goodness-of-fit index (GFI), and the root mean square error of approximation (RMSEA) along with its confidence interval. X^2^/df was used as an alternative to X^2^ to avoid distortions due to sample size, with values below 3 indicating a good fit. The other fit indices were analyzed on AMOS 23 software according to the established cutoff points [27,28]: for the CFI, values above 0.95 were deemed optimal, values above 0.90 were considered good, and for the RMSEA, values below 0.06 were regarded as optimal, while values below 0.08 were acceptable. All the statistical analyses were interpreted assuming a significance level of 5% (two-tailed).

## 3. Results

### 3.1. Descriptive Statistics of the Risk Variables Measured in Caregivers

The descriptive statistics for the anxiety, depression, stress, and subjective burden (ZBI-7) scores are shown in Table 2. All the variables’ obtained values of skewness and kurtosis were within the normal ranges.

### 3.2. Correlations Among Variables

The correlation matrix for the variables under study is given in Table 3. As can be seen, depression was significantly and positively correlated with stress (*r* = 0.713, *p* < 0.001), indicating that higher levels of depression were associated with higher levels of Stress.

Anxiety was significantly and positively correlated with stress (*r* = 0.733, *p* < 0.001) and depression (*r* = 0.760, *p* < 0.001), indicating that higher levels of anxiety were associated with higher levels of stress and depression.

Caregiver burden was significantly and positively associated with stress (*r* = 0.636, *p* < 0.001), depression (*r* = 0.550, *p* < 0.001), and anxiety (*r* = 0.542, *p* < 0.001), indicating that higher levels of burden were associated with higher levels of depression, anxiety, and stress.

Significant correlations were also observed as follows: level of disability and stress (*r* = 0.203, *p* = 0.001), depression (*r* = 0.174, *p* = 0.005), anxiety (*r* = 0.120, *p* = 0.039), and subjective burden (ZBI-7) (*r* = 0.232, *p* < 0.001), indicating that higher levels of disability were associated with increased stress, depression, anxiety, and perceived burden.

Hours of care was significantly and positively associated with stress (*r* = 0.0164, *p* = 0.005), subjective burden (ZBI-7) (*r* = 0.156, *p* = 0.005), and level of disability (*r* = 0.114, *p* = 0.035), indicating that higher hours of care are linked to higher levels of stress, perceived burden, and the severity of the disability.

### 3.3. Model SEM

We only considered the statistically significant variables in the prior inferential analyses. Table 4 gives the results based on the normality of the included variables and items by the commonly used maximum likelihood estimation method.

All the fit indices evaluated indicated that the empirical data aligned well with the theoretical model. The Chi-square value adjusted for degrees of freedom was 2.054 (X^2^/df), while both the CFI (0.955) and RMSEA (0.055, with a 90% confidence interval ranging from 0.046 to 0.065) also suggested a good fit.

**Figure 1:**
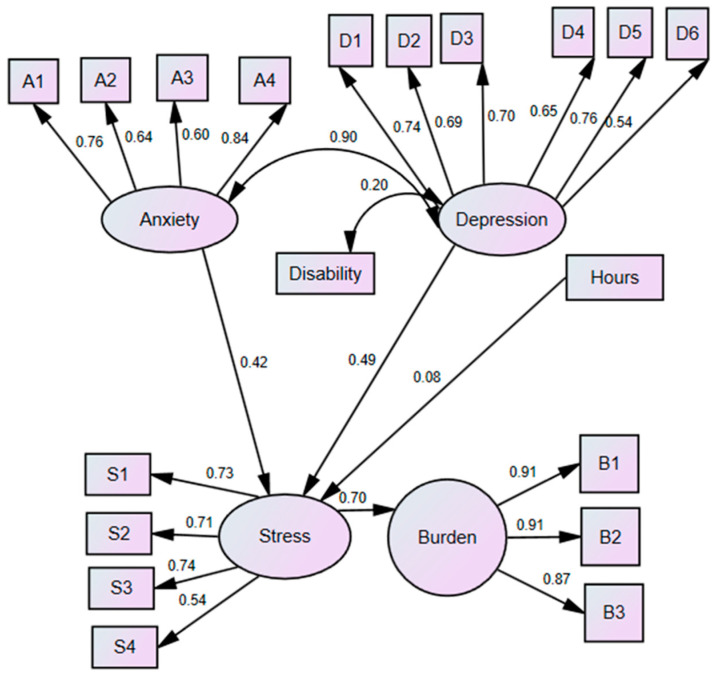
Standardized coefficients of the model of structural equations. Note: All parameters were statistically significant; errors and distributions not shown for the sake of clarity.

Figure 1 shows the model along with the standardized parameters obtained, i.e., the standardized regression weights. As expected, anxiety and depression were predictors of variance in stress (*p* = 0.006, *p* = 0.002, respectively), and stress was a predictor of variance in burden (*p* < 0.001), while anxiety and depression showed a statistically significant correlation (*p* < 0.001). However, the level of disability and hours of care were not predictors of the variance in caregiver burden (*β* = 0.23, *p* = 0.602, *β* = 0.075, *p* = 0.102, respectively), nor was the level of disability a predictor of stress (*β* = 0.075, *p* = 0.072)

## 4. Discussion

This study explored the functioning and practical implications of stress in informal caregivers by analyzing the implications of anxiety and depression for stress and the effect of stress on the caregiver burden. The relationships between anxiety, depression, the impact of the care recipient’s level of disability, and the hours of care were analyzed in the final endogenous variables.

It was assumed that both anxiety and depression of informal caregivers would negatively predict stress, so that caregivers with higher levels of depressive and anxious symptoms would experience more stress. The results fully supported this, as both depression and anxiety were found to be significant predictors of stress variance. These results are consistent with studies such as Del-Pino-Casado et al. [4], who found that caregivers with higher levels of anxiety and depression experienced higher levels of stress, and Christian et al. [29], who reported a significant correlation between depressive symptoms and caregiver stress, underscoring the relationship between mental health problems and stress among caregivers. From a theoretical perspective, the theory of perceived stress [30] suggests that anxiety and depression increase the perception of stress by affecting coping skills, making it difficult to manage the demands of caregiving. Recent studies, such as those by Kim and Schulz [31] and Losada et al. [32], have shown that caregivers with high levels of anxiety and depression face significantly higher levels of stress. This is due to a higher activation of the stress response system and a reduced ability to employ effective coping strategies, such as problem solving or seeking social support.

Hypothesis 1 predicted that caregiver stress would act as a risk factor for caregiver burden, with the expectation that greater stress would correlate with a greater burden. The results supported this hypothesis, revealing a positive and significant relationship, in line with previous studies that found that increased stress levels are directly associated with heightened caregiver burden, as in Baishya [33] and Lee et al. [34].

Several authors [35,36] confirm that chronic stress in caregivers significantly increases their sense of burden, affecting both their mental health and their ability to provide effective care. Furthermore, it was observed that interventions aimed at reducing stress in caregivers can mitigate perceived burden, reinforcing the importance of addressing this factor as a key component of caregiver well-being [37].

Hypothesis 2 hypothesized that caregivers’ anxiety and depression would be positively related, so that greater anxiety would be associated with greater depression. Our results confirmed this hypothesis and showed that high levels of anxiety often coincide with increased depressive symptoms, as in Thompson et al. [38], who found high levels of anxiety and depression among caregivers of individuals with chronic illnesses, and Lane et al. [39], who showed that the presence of anxiety intensifies depressive symptoms in caregivers.

Moreover, recent studies have further explored this relationship. García-Alberca et al. [40] found that caregivers of dementia patients who reported high levels of anxiety had a significantly higher risk of developing clinical depression, which underlines the importance of addressing both disorders in an integrated manner. Similarly, Pinquart and Sörensen [20] in their meta-analysis of caregivers’ mental health confirmed that anxiety and depression are comorbid in approximately 60% of cases, highlighting that caregiving stressors such as emotional overload and lack of social support act as common triggers for both disorders. On the other hand, Riffin et al. [15] identified that psychological interventions aimed at reducing anxiety in caregivers also significantly decrease depressive symptoms, which reinforces the idea that both disorders are interconnected and share underlying mechanisms.

Hypothesis 3 suggested that hours of care and level of disability would influence endogenous endpoints, although this hypothesis was not supported by the results, as the descriptive analyses showed that these variables did not correlate with the other variables. The theoretical model shown in Figure 1 reflects all these hypothesized relationships, and the empirical results showed an optimal fit, suggesting that the model is valid and replicable in future research, as both prosocial behavior and caregiver burden are statistically explained by the model. The lack of correlation between hours of care, level of disability, and endogenous variables could suggest the presence of moderating or mediating variables not considered in the current model. For example, perceived social support and caregiver resilience could play a crucial role in buffering the impact of these variables. Recent research has highlighted that social support, both emotional and instrumental, acts as a protective factor, mitigating the negative effects of caregiver burden and promoting psychological well-being [41]. Likewise, resilience, understood as the ability to adapt and recover from adverse situations, has been associated with lower levels of stress, anxiety, and depression in caregivers [42]. Therefore, it is plausible that these variables moderate or mediate the relationship between hours of caregiving, level of disability, and the outcomes studied, which could explain the lack of direct correlation observed in this study.

The proposed model shows an adequate fit to the empirical data, supported by the fit indices obtained. According to the criteria established in the literature, a value of X^2^/df of less than 3 indicates a good fit of the model [43], and in this study, a value of 2.054 was obtained, suggesting an adequate representation of the relationships between the variables. Furthermore, the CFI of 0.955 and RMSEA of 0.055 (with a 90% confidence interval between 0.046 and 0.065) meet the thresholds recommended by Hu and Bentler [27], who state that CFI ≥ 0.95 and RMSEA ≤ 0.06 reflect an optimal model fit to the observed data. These results are consistent with previous studies on informal caregivers, such as those of Kim and Schulz [31] and Losada et al. [32], where similar structural models have reported fit values within comparable ranges. In particular, research with similar samples has found RMSEA values between 0.05 and 0.07 and CFI values above 0.90, which reinforces the robustness of the present model. The sample size of 344 informal caregivers is sufficient for robust structural equation analysis (SEM). Following the recommendations of Boomsma and Hoogland [44], it is suggested that for models with moderate complexity, at least 200–300 cases are required to obtain stable estimates and avoid identification or overfitting problems. In this sense, the sample size used in this study exceeds the minimum recommended threshold, ensuring adequate statistical precision and allowing the results to be generalized to similar populations.

From a theoretical perspective, the fact that anxiety and depression significantly predict stress, and that stress in turn predicts caregiver burden, is consistent with classic models such as Lazarus and Folkman’s [30] Stress and Coping Model and the Caregiver Burden Model. These models have been validated in studies such as that of Pinquart and Sörensen [20], who found that psychological factors, such as anxiety and depression, play a key role in caregivers’ perception of stress.

On the other hand, the finding that the level of disability and hours of care do not significantly predict caregiver burden is consistent with studies such as Roth et al. [45], who argue that perceived burden is more related to emotional and psychological factors than to the objective amount of care provided.

### 4.1. Limitations and Future Directions

This study’s main limitation was its cross-sectional design, which prevented us from establishing definitive causal links between the variables studied. Future studies could benefit from a longitudinal design that would allow us to observe the dynamic interaction between anxiety, stress, depression, and caregiver burden over time. This approach would not only provide a deeper understanding of how these relationships evolve but also allow for more robust causal inferences. While useful, cross-sectional studies have a limited ability to establish causality and understand changes over time. A longitudinal approach could better elucidate how these factors influence each other and evolve at different points in the care process.

Another of its limitations is the restriction to the Comunidad Valenciana, which, along with the demographic and cultural uniformity of the caregiver sample, may limit the generalizability of the results to broader populations. Similar to other studies, such as Esparza et al. [46], which was also limited to the province of Valencia and published in a high-impact journal, this geographic focus ensures contextual relevance but may reduce external validity. Future research should aim to include greater demographic and cultural diversity in caregiver samples, encompassing variations in age, gender, ethnicity, socio-economic status, and cultural background. This would not only enhance the representativeness of the findings but also provide insights into how different cultural contexts influence caregivers’ levels of anxiety, depression, and stress. Such an approach could reveal unique protective or risk factors across cultures, contributing to the development of more personalized and effective interventions.

Finally, we consider that the development and evaluation of specific interventions aimed at reducing anxiety and depression among caregivers would be a promising line of research. These interventions would include psychological therapies, social support programs, stress management training, and self-help programs. Implementing randomized controlled trials to test the effectiveness of these interventions would provide strong evidence on the most beneficial strategies. Research should also explore the adaptability of these interventions to different cultural and demographic contexts, ensuring their relevance and effectiveness in diverse caregiver populations.

### 4.2. Practical Implications

The findings of this study have important practical implications for supporting informal caregivers, particularly in addressing the mental health challenges they face. Firstly, interventions aimed at reducing anxiety and depression among caregivers should be prioritized, as these mental health issues are significant predictors of stress, which in turn increases the caregiver’s burden. Psychological therapies, stress management training, and social support programs could be effective in alleviating these symptoms.

Secondly, the strong correlation between stress and caregiver burden suggests the need for stress-reduction strategies specifically designed for caregivers. These could include mindfulness training, relaxation techniques, and support groups designed to help caregivers manage their stress levels.

Thirdly, the relationship between anxiety and depression highlights the importance of comprehensive mental health screening of caregivers, allowing for early detection and intervention. Health professionals should routinely assess both their anxiety and depression to provide timely and holistic care.

Lastly, considering the lack of impact of hours of care and level of disability on the outcomes, future interventions should not only focus on these factors but also explore other variables that might affect caregivers’ stress and burden. Expanding research to include diverse demographic and cultural groups will help develop more inclusive and effective support strategies.

These findings are explained by Lazarus and Folkman’s (1984) [30] model of stress, which posits that burden arises when the demands of caregiving exceed the caregiver’s resources. Anxiety and depression limit those resources, increasing stress and thus burden. Reducing these symptoms improves coping skills and alleviates the impact of caregiving.

Furthermore, according to Cobb’s (1976) [47] theory of social support, perceived support protects against stress. This reinforces the need for specific networks and programs for caregivers. The weak influence of objective factors highlights the importance of psychosocial and cultural aspects, which are key to designing more effective and personalized support.

## 5. Conclusions

The findings of this study underline the central role of stress as a risk factor in informal caregiver burden. Anxiety and depression were confirmed to indirectly increase caregiver burden by increasing stress levels. Furthermore, although factors such as the level of disability of the care recipient and hours spent caring were not direct predictors of caregiver burden, the strong relationship between stress and burden suggests that interventions aimed at stress reduction could be key to improving caregivers’ well-being. These results reinforce the importance of psychological support and stress management strategies to mitigate the negative impact of prolonged caregiving on the mental health of informal caregivers.

## Figures and Tables

**Table 1:** Socio-demographic characteristics of the caregivers.

	n	%
Gender		
Female	202	58.7
Male	142	41.3
Hours of care		
1–56 h	298	86.6
57–112 h	15	4.4
113–168 h	31	9
Level of disability		
Low disability	60	17.4
Moderate disability	175	50.9
Severe disability	109	31.7
Ethnicity		
Caucasian	312	90.7
Other	15	4.4
Prefer nor to answer	17	4.9
Education Level		
No studies	10	2.9
Primary studies	61	17.7
Secondary studies	131	38.1
University studies	109	31.7
Post-university studies (Master’s, PhD, etc.)	26	7.6
Prefer not to answer	7	2.0
Marital status		
Single	59	17.2
Married	186	54.1
In relationship	64	18.6
Separated/Divorced	27	7.8
Widowed	3	0.8
Prefer not to answer	5	1.5

**Table 2:** Descriptive statistics of the protective variables measured in caregivers.

Variable	M	SD	Min.	Max.	Skewness	Kurtosis
Anxiety	5.25	3.67	0	20	0.644	0.129
Depression	5.67	4.04	0	21	0.754	0.149
Stress	8.05	3.72	0	20	0.413	0.194
Subjective Burden (ZBI-7)	19.14	6.57	2	35	0.161	−0.309

**Table 3:** Correlation analyses of the protective variables measured in caregivers.

		1	2	3	4	5	6
1. Stress	r	**-**					
	*p*-value	**-**					
	N	**-**					
2. Depression	r	0.713 **	**-**				
	*p*-value	0.000	**-**				
	N	344	**-**				
3. Anxiety	r	0.733 **	0.760 **	**-**			
	*p*-value	0.000	0.000	**-**			
	N	344	344	**-**			
4. Burden (ZBI-7)	r	0.636 **	0.550 **	0.542 **	-		
	*p*-value	0.000	0.000	0.000	-		
	N	344	344	344	-		
5. Level of disability	r	0.203 **	0.174 **	0.120 *	0.232 **	-	
	*p*-value	0.001	0.005	0.039	0.000	-	
	N	343	343	343	343	-	
6. Hours of care	r	0.164 **	0.091	0.090	0.156 **	0.114 **	-
	*p*-value	0.005	0.131	0.136	0.005	0.035	-
	N	344	344	344	344	343	-

Note. r = Pearson’s correlation coefficient. * *p* ≤ 0.05 (bilateral). ** *p* ≤ 0.01 (bilateral).

**Table 4:** Univariate skewness and kurtosis for items included in the model.

Variable	Skewness	Kurtosis
Anxiety		
A1	0.546	0.228
A2	1.023	0.680
A3	0.825	0.206
A4	0.770	−0.0051
Depression		
D1	0.617	0.333
D2	1.049	0.681
D3	0.847	0.179
D4	1.208	0.681
D5	0.515	−0.035
D6	0.809	0.004
D7	0.609	0.224
Stress		
S1	0.506	0.098
S2	0.414	0.145
S3	0.515	0.359
S4	0.653	0.511
Burden		
B1	0.039	−0.227
B2	0.194	−0.473
B3	0.302	−0.599

## Data Availability

The datasets presented in this article are not readily available because the data are part of an ongoing study or due.
